# Determining Mortality Rates Attributable to *Clostridium difficile* Infection

**DOI:** 10.3201/eid1802.101611

**Published:** 2012-02

**Authors:** Susy S. Hota, Camille Achonu, Natasha S. Crowcroft, Bart J. Harvey, Albert Lauwers, Michael A. Gardam

**Affiliations:** University Health Network, Toronto, Ontario, Canada (S.S. Hota, M.A. Gardam);; Ontario Agency for Health Protection and Promotion, Toronto (C. Achonu, N.S. Crowcroft);; University of Toronto, Toronto (N.S. Crowcroft, B.J. Harvey, M.A. Gardam);; Ministry of Community Safety and Correctional Services, Toronto (A. Lauwers)

**Keywords:** Clostridium difficile, mortality, mortality rates, deaths, death certificates, panel review, Ontario, Canada

## Abstract

To determine accuracy of measures of deaths attributable to *Clostridium difficile* infection, we compared 3 measures for 2007–2008 in Ontario, Canada: death certificate; death within 30 days of infection; and panel review. Data on death within 30 days were more feasible than panel review and more accurate than death certificate data.

*Clostridium difficile* infection (CDI) has emerged as a major health care–associated infection; incidence, hospitalizations, and mortality rates are increasing (*1**,**2*). Reported case-fatality rates are 6%–30% and seem to be rising (*3**,**4*). The reporting of CDI-associated deaths could be considered a quality indicator; however, the accuracy of death certificate data is questionable (*5*). We analyzed CDI deaths in 3 hospitals in Ontario, Canada, and compared 3 measures for attributing death to CDI: death certificate, death within 30 days of CDI, and a panel review process (considered the reference standard).

## The Study

From April 2007 through February 2008, as independent quality initiatives, 3 hospitals in Ontario reviewed deaths among patients with CDI infection. Patients were identified by using existing surveillance data. To calculate the time from CDI diagnosis to death, we compared date of death with date of onset of CDI symptoms or date of the positive *C. difficile* test result if symptom onset was unclear. For recurrent CDI, date of recurrent symptoms nearest to date of death was considered. Patients with suggestive recurrent symptoms but no laboratory confirmation were included in a further analysis in this study.

For panel review, clinical data and cause of death indicated on death certificate were anonymously summarized for each patient. Each case summary was reviewed by 3 physicians with varying levels of expertise with CDI. Panel members were asked to independently categorize their interpretation as follows: a) death was directly attributable to CDI; b) CDI strongly contributed to death; c) CDI somewhat contributed to death; d) death was unrelated to CDI; or e) information was insufficient to determine the role of CDI in the death. Hospital A was the first facility to undertake a panel review and included only 4 categories (category b was excluded). Feedback from reviewers at hospital A led to development of category b.

After individually classifying each death, reviewers participated in a panel discussion to achieve consensus. For analysis, the 5 categories were subsequently collapsed into 3: CDI directly caused or strongly contributed to death; CDI somewhat contributed to death or was unrelated to death; and information was insufficient to determine the role of CDI in the death.

The κ statistic was used to determine the level of agreement on cause of death between death certificate and panel review. The 3 categories listed above were compared with the following death certificate categories: CDI, enterocolitis, or toxic megacolon as primary or contributory cause of death; death unrelated to CDI; and missing information.

The percentage agreement of death within 30 days of CDI and panel review consensus was calculated by using combined data from hospitals A and B. Hospital C was excluded from this analysis because only patients who died within 30 days of CDI diagnosis were included in that review. At hospital B, because data on individual physician assignment of categories were available, inter-rater reliability was analyzed by using the Fleiss κ statistic.

CDI was diagnosed for 501 patients, and 188 CDI patients died. Of these, 120 (64%) patients died within 30 days of CDI. The 30-day case-fatality ratios for hospitals A, B, and C were 20% (25/124), 35% (62/177), and 16% (33/200), respectively.

Panel reviews were conducted for all 31 in-hospital deaths in hospital A, 90 deaths in Hospital B, but only 30 of the deaths that occurred within 30 days of CDI diagnosis in hospital C. Among the 151 deaths included in the panel review process, CDI directly caused or strongly contributed to the death for 101 (67%) and somewhat contributed or was unrelated to death for 49 (32%). For 1 patient, information was insufficient for determining cause of death. Where data were available (hospital B), inter-rater reliability among panel members was satisfactory (κ = 0.71, 95% CI 0.59–0.83).

According to death certificate data, CDI was the primary cause of death for 7 (5%) patients and a contributory cause of death for 44 (29%). Of the 101 deaths classified by panel review as strongly attributable to CDI, 37 were classified by death certificate as having CDI as primary/secondary cause (Table 1). When panel review data were compared with death certificate data, κ was 0.07 (95% CI 0.05–0.20), indicative of poor agreement. The exclusion of hospital A, where deaths were originally classified into only 4 categories, did not alter the observed κ scores (κ = 0.04, 95% CI 0.09–0.17).

**Table 1:** Proportion of deaths attributable to CDI, by panel review coding and death certificate classification, Ontario, Canada, April 2007–February 2008*

Reference category	Death certificate, no. (%)				Panel review, no.†
	CDI as primary cause of death	CDI as primary or secondary cause of death	CDI as unrelated cause of death	Missing information	
Directly/strongly attributable to CDI	6 (6)	37 (37)	61 (60)	3 (3)	101
Somewhat/unrelated to CDI	1 (2)	14 (29)	35 (71)	0	49
Insufficient information	0	0	1 (100)	0	1
Total	7 (5)	51 (34)	97 (64)	3 (2)	151

*CDI, *Clostridium difficile* infection. †Categories determined by panel review. Because panel review was considered the reference standard, panel review percentages = 100%.

To compare the proportion of in-hospital deaths within 30 days with results of the panel review, we used only data from hospitals A and B because hospital C data only included patients who had died within 30 days. The panel concluded that CDI directly or strongly contributed to death within 30 days of onset for 80% (63/79) of patients (Table 2). If cases suggestive of recurrent CDI (not confirmed by testing) were included, this percentage rose to 86% (68/79). When panel review was used as the reference standard, the sensitivity of death within 30 days of CDI onset was 80%, specificity 41%, and positive predictive value 72%.

**Table 2:** Proportion of deaths within 30 days after CDI, by panel review coding, and hospital, Ontario, Canada, April 2007–February 2008*

Reference category†	No. (%) deaths for hospital A within 30 d after		Panel review, no.	No. (%) deaths for hospital B within 30 d after		Panel review, no.	No. (%) deaths for hospitals A and B within 30 d after		Panel review, no.
	Toxin-positive result	Clinical onset‡		Toxin-positive result	Clinical onset‡		Toxin-positive result	Clinical onset‡	
Directly/strongly attributable to CDI	17 (94)	17 (94)	18	46 (75)	51 (84)	61	63 (80)	68 (86)	79
Somewhat/ unrelated to CDI	8 (62)	8 (62)	13	16 (57)	18 (64)	28	24 (59)	26 (63)	41
Insufficient information	–	–	–	0	0	1	0	0	1
Total	25 (81)	25 (81)	31	62 (69)	69 (77)	90	87 (72)	94 (78)	121

*CDI, *Clostridium difficile* infection. †Categories determined by panel review. Because panel review was considered the reference standard, panel review percentages = 100%. ‡Because recurrent CDI is not always supported by laboratory or pathology confirmation, those patients with recurrent symptoms suggestive of CDI but no toxin confirmation were included by using the date of clinical onset of symptoms nearest to the date of death.

## Conclusions

Agreement between causes of deaths categorized by review panel and causes listed on death certificates is poor. Ontario’s vital statistics system currently codes only 1 cause of death, limiting the ability to identify deaths for which CDI might have been a contributing cause. These shortcomings suggest that death certificate data may be inaccurate for assigning CDI-attributable death.

Panel review of all deaths is an alternative approach that would enable clinical analysis of the circumstances surrounding the death. A panel review reduces individual reviewer bias; however, while arguably the most accurate method of determining cause of death other than autopsy, it is not feasible for wide-scale public reporting.

Our study supports the use of death within 30 days as a marker for CDI-attributable death because 80% of deaths identified by panel review as being directly or strongly attributable to CDI occurred within 30 days of diagnosis. This percentage increased to 86% if clinical recurrences were included. Compared with panel review, death within 30 days had reasonable sensitivity (80%) and positive predictive value (72%) but, as expected, was not specific (41%). Capturing data on death within 30 days would be more feasible than panel review and more accurate than death certificate data. However, data on death within 30 days could not be used to determine the contribution of CDI to any patient’s death.

Our study has a few limitations. Because data were derived from 3 hospitals that undertook reviews for different purposes, the slight differences in inclusion criteria and death categorizations necessitated subanalyses for the death within 30 days comparison and the collapsing of the categorizations into 3 groups. Data quality also has inherent problems associated with retrospective chart audits because of limitations in the documentation of clinical events. Finally, we acknowledge that categorization of deaths by panel review is a subjective process based on interpretation of the clinical case summaries and expert opinion.

Our findings suggest that cause of death on death certificate is an inaccurate measure of death attributable to CDI. However, death from CDI within 30 days should be considered a feasible measure for the purposes of aggregate public reporting.
